# Neopterin and kynurenic acid as predictors of stroke recurrence and mortality: a multicentre prospective cohort study on biomarkers of inflammation measured three months after ischemic stroke

**DOI:** 10.1186/s12883-021-02498-w

**Published:** 2021-12-08

**Authors:** Katinka Nordheim Alme, Arve Ulvik, Torunn Askim, Jörg Assmus, Tom Eirik Mollnes, Mala Naik, Halvor Næss, Ingvild Saltvedt, Per-Magne Ueland, Anne-Brita Knapskog

**Affiliations:** 1 Institute of Clinical Medicine (K1), University of Bergen, Bergen, Norway; 2 Department of Internal Medicine, Haraldsplass Deaconess Hospital, Bergen, Norway; 3 Bevital AS, Bergen, Norway; 4 Department of Neuromedicine and Movement Science, Faculty of Medicine and Health Science, NTNU-Norwegian University of Science and Technology, Trondheim, Norway; 5 Centre for Clinical Research, Haukeland University Hospital, Bergen, Norway; 6 Department of Immunology, Oslo University Hospital and University of Oslo, Oslo, Norway; 7 Research Laboratory, Nordland Hospital, Bodø, and K.G. Jebsen TREC, University of Tromsø, Tromsø, Norway; 8 Centre of Molecular Inflammation Research, NTNU-Norwegian University of Science and Technology, Trondheim, Norway; 9 Department of Clinical Science (K2), University of Bergen, Bergen, Norway; 10 Department of Neurology, Haukeland University Hospital, Bergen, Norway; 11 Centre for age-related medicine, Stavanger University Hospital, Stavanger, Norway; 12 Department of Geriatrics, Clinic of internal medicine, St Olavs Hospital, Trondheim University Hospital, Trondheim, Norway; 13 Department of Geriatric Medicine, Oslo University Hospital. Ullevaal, Oslo, Norway

**Keywords:** Sedentary behaviour, Inflammation, Immune modulation, Vascular disease, Kynurenine pathway, Stroke

## Abstract

**Background:**

Chronic low-grade inflammation is associated with both ischemic stroke and sedentary behaviour. The aim of this study was to investigate the predictive abilities of biomarkers of inflammation and immune modulation associated with sedentary behaviour for ischemic stroke recurrence and mortality in a stroke population.

**Methods:**

Patients admitted to hospital for acute stroke were recruited to the prospective multicentre cohort study, the Norwegian Cognitive Impairment After Stroke (Nor-COAST) study, from May 2015 until March 2017. Patients with ischemic stroke, blood samples available from the three-month follow-up, and no stroke recurrence before the three-month follow-up were included. Serum was analysed for C-reactive protein (CRP) with high-sensitive technique, and plasma for interleukin-6 (IL-6), neopterin, pyridoxic acid ratio index (PAr-index: 4-pyridoxic acid: [pyrioxal+pyridoxal-5′-phosphate]) and kynurenic acid (KA). Ischemic stroke recurrence and death were identified by the Norwegian Stroke Registry and the Cause of Death Registry until 31 December 2018.

**Results:**

The study included 354 patients, 57% male, mean age 73 (SD 11) years, mean observation time 2.5 (SD 0.6) years, and median National Institute of Health Stroke Scale of 0 (IQR 1) at three months. CRP was associated with mortality (HR 1.40, CI 1.01, 1.96, *p* = 0.046), and neopterin was associated with the combined endpoint (recurrent ischemic stroke or death) (HR 1.52, CI 1.06, 2.20, *p* = 0.023), adjusted for age, sex, prior cerebrovascular disease, modified Rankin Scale, and creatinine. When adding neopterin and KA to the same model, KA was negatively associated (HR 0.57, CI 0.33, 0.97, *p* = 0.038), and neopterin was positively associated (HR 1.61, CI 1.02, 2.54, *p* = 0.040) with mortality. Patients with cardioembolic stroke at baseline had higher levels of inflammation at three months.

**Conclusion:**

Neopterin might be a valuable prognostic biomarker in stroke patients. The use of KA as a measure of anti-inflammatory capacity should be investigated further.

**Trial registration:**

The study was registered at Clinicaltrials.gov (NCT02650531). First posted on 08/01/2016.

**Supplementary Information:**

The online version contains supplementary material available at 10.1186/s12883-021-02498-w.

## Background

Ischemic stroke is associated with chronic low-grade inflammation, and there is a need for more knowledge about mechanisms and management [1–4]. Prognosis, outcomes, and management of stroke depends on stroke subtype, defined by the assumed aetiology of atherosclerosis, cardioembolism, or small vessel disease [5, 6]. In the context of inflammation, atherosclerosis is the most studied subtype, but inflammation has been found to contribute to all three categories [7–10]. Despite the evidence, clinical trials using drugs targeting inflammation have so far been inconclusive, and there are to date no established preventive treatment strategies that target inflammation in particular [4, 11], possibly related to the challenge of obtaining an optimal balance between potential benefits and an increased risk of (fatal) infections [4]. Obviously, treatment strategies that minimise severe side effects are desired.

Reducing chronic low-grade inflammation by targeting sedentary behaviour has shown promising results [12]. Still, there is a need for valid biomarkers associated with inflammation with explanatory and predictive properties for outcomes like vascular disease and mortality [3]. In a prior study, we investigated blood biomarkers of inflammation and immune modulation in a stroke population measured 3 months after the acute ischemic event and their association to objectively measured sedentary time. We identified the biomarkers C-reactive protein (CRP), interleukin-6 (IL-6), neopterin, and the pyridoxal acid ratio-index (PAr-index) to be positively associated, and kynurenic acid (KA) to be negatively associated with objectively measured sedentary behaviour [13]. CRP [14], IL-6 [15], neopterin [16, 17] and PAr-index [18] are inflammatory biomarkers associated with vascular disease. KA is part of a negative feedback loop inducing immune tolerance [19, 20]. Hence, even though it will rise in response to inflammation, this response is believed to be beneficial for disease progression during an inflammatory state [20]. The association to vascular disease progression is unclear.

In this study, we investigated the associations between biomarkers of inflammation and stroke recurrence and all-cause mortality. Finally, given the above-mentioned clinical importance of the stroke subtypes, we also investigated the association to the stroke subtype at baseline.

## Material and methods

### Subjects

**Fig. 1:**
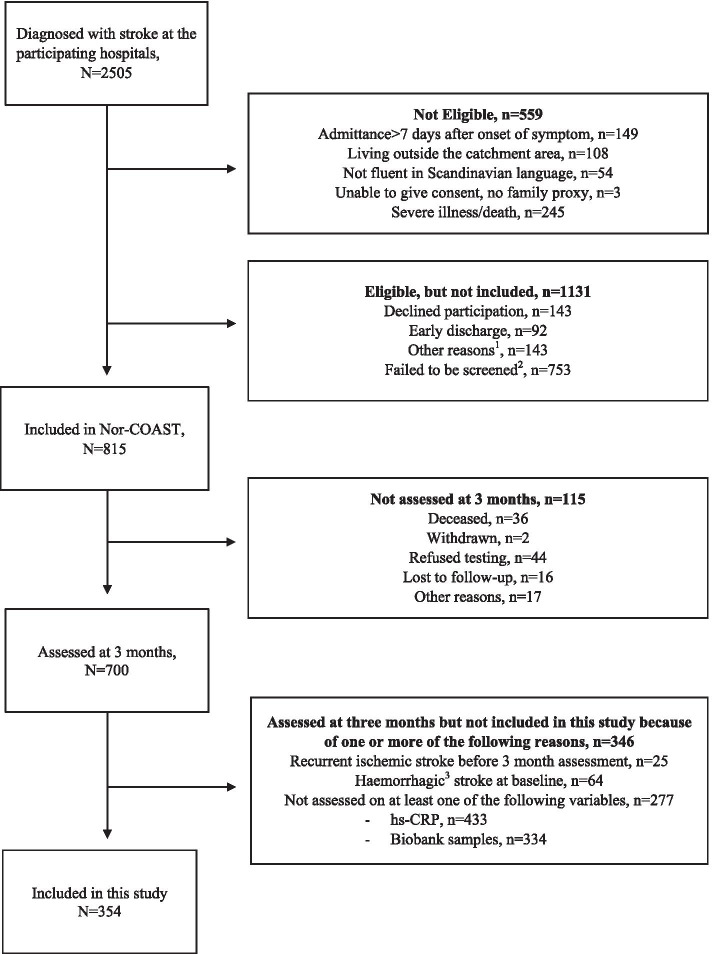
Patient selection. ^1^ Other reasons: i.e.: delirious patient, hearing, uncertainty about the diagnosis, multi morbid, nursing home resident. ^2^ Failed to screen: infrastructure on the ward, vacation/weekends. ^3^ Not haemorragic transformation

The patients were included in the prospective multicentre cohort study, the Norwegian Cognitive Impairment After Stroke (Nor-COAST) study, at admission for acute stroke between May 2015 and March 2017. The inclusion criteria were 1) hospital admission to one of the five participating hospitals, 2) acute stroke following the World Health Organisation definition or finding of acute stroke on imaging 3) being able to communicate in one of the Scandinavian languages, 4) above 18 years of age, 5) living in the catchment area. Patients were excluded if they had a life expectancy of less than 3 months. Details about the Nor-COAST population are described in prior studies [21, 22]. For this study, only patients with ischemic stroke at baseline who participated at the three-month follow-up and with data on at least one of the relevant biomarkers were included. To avoid the acute inflammatory response associated with the acute stroke event, we chose the three-month follow-up after the acute stroke as the baseline for this study, assuming the inflammatory biomarkers were not affected by the acute event at this time point (Fig. 1) [23].

### Clinical data

Demographic data and information about stroke properties, namely lesion type and stroke subtype, and stroke risk factors, were collected at baseline. Waist circumference, smoking status, stroke severity and functional outcomes were assessed at the three-month follow-up. Stroke severity was measured using the National Institute of Health Stroke Scale (NIHSS), global function by use of the modified Rankin Scale (mRS), and basic activities of daily living (ADL) by the Barthel Index (BI). The diagnosis of diabetes mellitus was defined at baseline by medical history and/or medication use (Anatomical therapeutical chemical Classification (ATC): A10) and/or HbA1c ≥6.5% at baseline. Hypercholesterolemia at baseline was defined by medication use (ATC: C10) and/or total cholesterol > 6.2 mmol/L and/or LDL ≥ 4.1 mmol/L at baseline. Hypertension at baseline was defined by medication use (ATC: C02, C03, C04, C07, C08, C09).

### Laboratory analyses

At the three-month follow-up, non-fasting blood samples were collected for routine analysis and for storage in the biobank.

*Routine clinical–chemical analyses*: serum C-reactive protein (CRP) – measured using a high-sensitive technique (mg/L) – and serum creatinine (μmol/L) were analysed at the local laboratories of the inclusion centres.

*Sample collection of research analyses:* Aliquots of serum and plasma were immediately frozen at − 80 °C, and sent on dry ice to BioBank1, Central Norway Regional Health Authority for storage. The inflammatory biomarkers were analysed in 2019. Two aliquots of plasma were used, one for each of the two laboratories. The samples were thawed only once. The selection of biomarkers for this study was based on prior findings [13] and included CRP (mg/L), interleukin-6 (IL-6, pg/ml), neopterin (nmol/L), 4-pyridoxic acid (nmol/L), pyridoxal, pyridoxal 5′-phosphate (nmol/L), and kynurenic acid (KA, nmol/L). The pyridoxic acid ratio index – PAr-index: 4-pyridoxic acid (pyrioxal+pyridoxal 5′-phosphate) [24] was calculated. The cytokines were analysed at Research Laboratory Nordland Hospital using the Bio-Plex technology kits obtained from Bio-Rad Laboratories Inc., Hercules, CA.. The other biomarkers were analysed as part of analytic platform D at Bevital A/S (Bergen, Norway) by liquid chromatography/tandem mass spectrometry. Because of the sample size, IL-6 was analysed using multiple trays. The upper and lower detection limit varied among the trays according to the standard curve. There were no values above or below the detection limits for IL-6. For the biomarkers from BeVital AS (neopterin, kynurenic acid, and the B6 vitamers), the performance of the method has been published previously [25]. There were no values out of range.

### Outcomes

Recurrent ischemic stroke was identified by the Norwegian Stroke Registry. The coverage was 84–87% in the period from 2015 to 2018, and stroke was defined according to the WHO’s definition [26]. Death was identified by the Norwegian Cause of Death Registry, which has a general coverage of 98% of deaths of Norwegian citizens within the country or abroad [27]. The data extraction was performed on 31 December 2018.

Patient outcomes were defined based on the first event: recurrent ischemic stroke, death or no event. Some of the patients died during the follow-up period after first having a stroke recurrence. In the regression analyses, these patients were included in both groups. A third outcome was defined as the composite of recurrent ischemic stroke and death. The patients with both outcomes were counted only once.

### Statistics

For the baseline and three-month descriptions, patients were stratified by first event and analysed using the chi-squared test for categorical variables (numbers and percentages), one-way ANOVA for continuous variables, and Kruskal–Wallis H-test for the continuous variables that were not normally distributed. Data were presented as means with standard deviations (SD) and medians with interquartile range (IQR).

One-way ANOVA were used for the analyses of laboratory values according to stroke subtype at baseline. The subgroup “other determined” was excluded.

The associations between the individual biomarkers and the outcomes stroke, mortality and the composite endpoint were analysed using Cox regressions. The start of the follow-up time was defined as the date of the three-month visit to the hospital. For the outcome “recurrent ischemic stroke”, an additional analysis using a competing-risk Cox regression based on the method of Fine and Grey [28] as an alternative to the regular Cox regression was used [29], with death as a competing outcome for ischemic stroke. All the biomarkers were right-skewed. After log-transformation, regression residuals approached normal distribution. The variables were standardised, and the hazard ratios are given per standard deviation change of the variable value. To account for potential confounders, we added modified Rankin scale and prior cerebrovascular disease as independent variables, because they have been found to be associated with both stroke recurrence [30] and with inflammation (mRS [31], prior cerebrovascular disease (before the baseline stroke in this study) [32]). We also added age and sex. Creatinin was included because some of the biomarkers had renal clearance (Model 1). As the blood samples were taken at the three-month follow-up, we used the results of the mRS recorded at this time point. To account for the different pathophysiology of the stroke subtypes, we did a second analysis where we added the Trial of Org 10,172 in Acute Stroke Treatment (TOAST) criteria to the model (Model 2), defining five stroke subtypes (large artery atherosclerosis, cardioembolic, cerebral small vessel disease, other determined, undetermined). The stroke subtype “other determined” is used when the cause of stroke is determined (i.e. dissection, vasculitis). This group was excluded from this analysis because of low sample size. Because KA is part of a feedback loop in response to inflammation, a third regression model including both KA and neopterin in addition to the same covariates as in Model 1 was made.

The significance level was set to 0.05, but *p*-values > 0.01 should be evaluated with caution due to a high number of tests. The analyses were performed in STATA/SE 16.1 (Stata Corp LLC, College Station, TX, USA).

## Results

### Patient characteristics and laboratory values

Of the 700 patients attending the three-month follow-up, 354 were included in this sub-study. The remaining 346 patients were excluded due to at least one of the following reasons: stroke before 3 months (*n* = 25), haemorrhagic stroke at baseline (*n* = 64), or no relevant biomarker available (*n* = 277).

The mean follow-up time from baseline for the whole group was 2.5 (0.6) years, and the mean time-to-event was 1.5 (1.0) and 1.6 (0.7) years for stroke and death, respectively. From the three-month follow-up and throughout the follow-up time, 16 (4.5%) patients had ischemic stroke recurrence. In total, 28 (7.9%) patients died, and 25 (7.1%) of them died without being registered as having stroke recurrence.

The baseline and three-month data are presented in Table 1, stratified by the first event. The patients who died were older compared to those with no event. Patients with no events had a higher Barthels Index (p 0.007) and a lower mRS (*p* < 0.001). There were overall differences between the groups in the presence of prior ischemic stroke (p < 0.001), prior coronary artery disease (*p* = 0.007), and atrial fibrillation at baseline (*p* = 0.034), the patients experiencing no events showing the lowest prevalence. There were overall differences between the groups in the values for IL-6, neopterin, and the PAr-index, where the patients who died had the highest values.

**Table 1:** Baseline and three-month characteristics according to the first event after three-month follow-up

	All ***N*** = 354	New IS ***N*** = 16	Death ***N*** = 25^**1**^	No event ***N*** = 313	***P-***value
Age^2,3^, mean years (SD)	73 (11)	73 (10)	82 (9)	73 (11)	< 0.001
Male^4^ sex, n (%)	201 (57)	11 (69)	16 (64)	174 (56)	0.439
Observation time^3^, mean years (SD)	2.5 (6)	1.5 (1.0)	1.6 (0.7)	2.6 (0.5)	< 0.001
**Baseline**					
TOAST^4^ subtype^6^, n (%)					0.661
Large artery	30 (9)	1 (6)	0	29 (10)	
Cardioembolic	72 (21)	5 (31)	6 (24)	61 (20)	
Small vessel disease	83 (24)	4 (25)	6 (7)	73 (24)	
Other determined	10 (3)	1 (6)	0	9 (3)	
Undetermined	128 (43)	5 (31)	13 (52)	128 (43)	
Prior IS^4^, n (%)	72 (20)	7 (44)	13 (52)	52 (17)	< 0.001
Prior CHD^4^, n (%)	61 (17)	3 (19)	10 (40)	48 (13)	0.007
Atrial fibrillation^4^, n (%)	89 (25)	6 (37)	11 (44)	72 (23)	0.034
Hypertension^4^, n (%)	202 (57)	11 (69)	19 (76)	172 (60)	0.077
Hypercholesterolemia^4^, n (%)	178 (50)	8 (50)	16 (64)	154 (46)	0363
Diabetes mellitus^4^, n (%)	60 (17.0)	0	6 (24)	54 (17)	0.124
**Three-months**					
NIHSS^5^ median (IQR)	0 (1)	1 (1)	0.5(3)	0 (1)	0.122
BI^5^ median (IQR)	100 (0)	100 (0)	95 (17)	100 (0)	0.017
mRS^5^ median (IQR)	1 (1)	2 (1)	3 (2)	1 (1)	< 0.001
Waist^2, 3^, mean cm (SD)	95 (13)	89 (15)	98 (15)	95 (13)	0.137
Current smoking^2, 4^, n (%)	31 (9)	1 (7)	4 (17)	26 (9)	0.597
Laboratory measures^2,5^					
CRP, median (IQR), *n* = 267	1.6 (2.9)	1.1 (5.3)	3.0 (26.5)	1.6 (2.9)	0.053
IL-6, median (IQR), *n* = 334	4.6 (4.5)	4.8 (4.1)	6.1 (6.1)	4.2 (4.2)	0.038
Neopterin, median (IQR), n = 334	16 (10)	17 (12)	24 (17)	16 (9)	< 0.001
PAr-index, median (IQR), n = 334	0.62 (0.46)	0.57 (0.80)	1.0 (0.8)	0.60 (0.42)	0.002
Kynurenic acid, n = 334	58 (30)	56 (35)	65 (52)	59 (29)	0.522

IS = ischemic stroke. *TOAST = *Trial of Org 10,172 in Acute Stroke Treatment. CHD = coronary heart disease. NIHSS=National Institute of Stroke Scale. IQR = interquartile range. BI = Barthels Index. mRS = modified Rankin Scale. CRP = C-reactive protein. IL-6 = Interleukin-6. PAr-index = pyridoxic acid ratio-index = 4-pyridoxic acid:(pyridoxal+pyridoxal 5′-phosphate)

^1^Additionally, three patients died after having recurrent strokes

^2^At three months

^3^The one-way ANOVA was used for the continuous variables

^4^The chi-squared test was used for the categorical variables

^5^For the continuous variables with non-normal distribution, the non-parametric Kruskal–Wallis H-test was used

^6^TOAST subtype of index stroke

### Stroke subtype

The biomarker levels by stroke subtype at baseline are presented in Table 2. There was a significant overall difference between the groups in the level of CRP, IL-6, and neopterin. Patients with cardioembolic stroke at baseline showed the highest values.

**Table 2:** Biomarker values at three-month follow-up by ischemic stroke subtype at baseline^1^

	Atherosclerosis (N = 26)	Cardioembolic (***N*** = 62)	CSVD (***N*** = 75)	Unknown (***N*** = 141)	***P***-value
CRP^2^, mean (SD)	1.93 (3.29)	3.06 (3.78)	1.88 (2.56)	1.58 (3.71)	0.032
IL-6, mean (SD)	3.71 (1.95)	5.70 (2.18)	3.97 (2.18)	4.26 (2.39)	0.038
Neopterin, mean (SD)	16.0 (1.5)	20.3 (1.7)	16.0 (1.4)	16.0 (1.5)	0.003
PAr, mean (SD)	0.66 (1.77)	0.73 (1.73)	0.60 (1.73)	0.60 (1.56)	0.087
KA, mean (SD)	59.7 (1.5)	67.4 (1.4)	59.7 (1.5)	57.4 (1.5)	0.093

^1^The category “other determined” was excluded because of low sample size. The analyses were conducted on log-transformed variables, and the values were transformed back after analysis

^2^For CRP: Atherosclerosis: *N* = 20, cardioembolic: *N* = 46, CSVD: *N* = 50, Unknown: *N* = 100. CSVD=cerebral small vessel disease. CRP = C-reactive protein IL-6 = Interleukin-6. PAr = pyridoxic acid ratio-index = 4-pyridoxic acid:(pyridoxal+pyridoxal 5′-phosphate). KA = kynurenic acid. The analyses were done using a one-way ANOVA

### Regression analyses

The crude and adjusted hazard ratios (HR) for the outcome’s ischemic stroke recurrence, death, and the composite endpoint of stroke and death, investigated for each of the biomarkers individually, are presented in Table 3. We did not find any associations with stroke recurrence. In the crude analyses, all biomarkers except for KA were significantly associated with the risk of death or the composite endpoint. In the adjusted analyses, we found that CRP was associated with death (Model 1: HR 1.40, (CI 1.01, 1.96), *p* = 0.046) and neopterin with the combined endpoint (Model 1: HR 1.52, (CI 1.06, 2.20), *p* = 0.023. Model 2: HR 1.55, (CI 1.06, 2.27), *p* = 0.025).

**Table 3:** Crude and adjusted Cox regressions of the association between the individual biomarkers and ischemic stroke recurrence, death and the composite endpoint (ischemic stroke recurrence and death)

		Crude			Model 1			Model 2		
		HR	CI	*P*	HR	CI	*p*	HR	CI	*P*
**Recurrent ischemic stroke, N = 16**										
	CRP^1)^	0.90	(0.44, 1.83)	0.772	0.86	(0.41, 1.77)	0.674	0.92	(0.44, 1.90)	0.816
	IL-6	1.11	(0.67, 1.86)	0.686	1.19	(0.52, 1.97)	0.578	1.10	(0.30, 2.02)	0.754
	Neopterin	1.34	(0.85, 2.11)	0.209	1.45	(0.82, 2.57)	0.201	1.39	(0.80, 2.41)	0.244
	PAr	1.16	(0.71, 1.90)	0.553	1.13	(0.61, 2.10)	0.692	1.24	(0.67, 2.31)	0.494
	KA	0.91	(0.55, 1.50)	0.710	0.82	(0.41, 1.61)	0.563	0.89	(0.43, 1.86)	0.761
**Death**^**2**^**,** ***N*** **= 28**										
	CRP^1)^	1.81	(1.29, 2.53)	0.001	1.40	(1.01, 1.96)	0.046	1.41	(1.00, 1.99)	0.053
	IL-6	1.74	(1.20, 2.51)	0.003	1.34	(0.87, 2.07)	0.181	1.36	(0.87, 2.12)	0.173
	Neopterin	1.93	(1.43, 2.61)	< 0.001	1.44	(0.89, 2.33)	0.136	1.55	(0.91, 2.64)	0.104
	PAr	1.69	(1.15, 2.49)	0.008	1.11	(0.68, 1.82)	0.666	1.13	(0.67, 1.90)	0.641
	KA	1.12	(0.75, 1.66)	0.577	0.64	(0.38, 1.07)	0.088	0.69	(0.41, 1.14)	0.145
**Stroke and death,** ***N*** **= 41**										
	CRP^1)^	1.59	(1.16, 2.18)	0.004	1.27	(0.92, 1.74)	0.140	1.27	(0.93, 1.74)	0.135
	IL-6	1.47	(1.08, 2.02)	0.016	1.23	(0.85, 1.77)	0.274	1.23	(0.85, 1.77)	0.275
	Neopterin	1.79	(1.39, 2.31)	< 0.001	1.52	(1.06, 2.20)	0.023	1.55	(1.06, 2.27)	0.025
	PAr	1.62	(1.19, 2.22)	0.002	1.28	(0.86, 1.90)	0.224	1.34	(0.89, 2.01)	0.162
	KA	1.27	(0.58, 2.79)	0.555	0.55	(0.19, 1.54)	0.252	0.60	(0.21, 1.71)	0.339

HR = hazard ratio. CI = confidence interval. CRP = C-reactive protein. IL-6 = Interleukin-6. PAr = PAr-index = pyridoxic acid ratio-index = 4-pyridoxic acid:(pyridoxal+pyridoxal-5`-phosphate). KA = kynurenic acid. Each biomarker was investigated individually

Model 1: Age, sex, prior cerebrovascular disease, modified Rankin Scale at 3 months, creatinine

Model 2: As in model 1 + TOAST-classification at baseline (TOAST = Trial of Org 10,172 in Acute Stroke Treatment)

^1^For CRP, *N* = 212, 8 and 13 for all, recurrent IS and death, respectively

^2^Includes both those with and without ischemic stroke recurrence prior to death

In the additional competing risk regression analyses, where we used death as a competing event for stroke recurrence, the risk of ischemic stroke recurrence did not change significantly (see Supplementary Table 1).

The regression analyses, including both KA and neopterin in addition to the variables in Model 1, are shown in Table 4. KA was negatively associated with mortality (HR 0.57, (0.33, 0.97), 0.038), and neopterin was positively associated with mortality (HR 1.61, (1.02, 2.54), 0.040). Neopterin was also associated with the composite endpoint (HR 1.59, (1.11, 2.27), 0.011).

**Table 4:** Adjusted Cox regression (Model 1) of the combined contributions of kynurenic acid and neopterin in stroke, mortality and the composite endpoint (stroke and death)

	Stroke recurrence			Death			Stroke and death		
	HR	CI	p	HR	CI	p	HR	CI	p
KA	0.78	(0.39, 1.57)	0.492	0.57	(0.33, 0.97)	0.038	0.72	(0.46, 1.10)	0.131
Neopterin	1.48	(0.83, 1.57)	0.177	1.61	(1.02, 2.54)	0.040	1.59	(1.11, 2.27)	0.011
Age	0.98	(0.51, 2.61)	0.949	1.11	(0.60, 2.06)	0.746	1.09	(0.68, 1.76)	0.720
Sex	1.39	(0.73, 2.66)	0.314	1.61	(1.00, 2.62)	0.052	1.43	(0.97, 2.10)	0.071
Prior CeVD	1.37	(0.87, 2.17)	0.175	1.56	(1.10, 2.22)	0.012	1.44	(1.08, 1.91)	0.012
mRS	0.97	(0.46, 2.05)	0.945	3.25	(1.77, 5.96)	< 0.001	2.24	(1.40, 3.57)	0.001
Creatinine	1.31	(0.20, 3.38)	0.583	2.23	(1.12, 4.42)	0.022	1.65	(0.93, 2.92)	0.085

The analyses includes KA, neopterin and Model 1: Age, sex, prior cerebrovascular disease, modified Rankin Scale at three months, creatinine

HR = hazard ratio. CI = confidence interval. KA = kynurenic acid. CeVD = cerebrovascular disease. mRS = modified Rankin Scale

## Discussion

In this study, we showed that higher levels of C-reactive protein (CRP) measured 3 months after acute ischemic stroke were associated with an increased risk of death. Neopterin was associated with the composite endpoint of stroke and death. In a model including both neopterin and kynurenic acid, neopterin and kynurenic acid were associated with an increased and a reduced risk of death, respectively. Furthermore, patients with cardioembolic stroke at baseline and those who died during follow-up had higher levels of pro-inflammatory biomarkers at 3 months.

### Stroke recurrence

We did not find any association between inflammatory biomarkers and stroke recurrence. This could be due to low sample size, the relatively short time of follow-up, or not all recurrent strokes being clinically acknowledged leading to a statistical type II error. The role of inflammation in stroke recurrence, investigated by traditional biomarkers such as IL-6 and CRP, has been well established [14, 15, 33, 34]. The novel biomarkers; neopterin, PAr-index, and KA, are less studied. Neopterin has shown to predict future coronary events [16, 17] and stroke recurrence [35]. Neopterin has also been associated with complex carotid plaques in patients with stable angina pectoris [36], with the extent of cerebral small vessel disease (CSVD) in stroke patients [37] and with the presence, but not the future risk, of atrial fibrillation [38]. The PAr-index has been associated with future stroke in a population study [18] but has never been studied in a stroke population nor in the context of stroke recurrence. The role of KA in stroke recurrence is not clear, but KA has been found to be associated with coronary events [39, 40] and aortic stiffness, which in turn is associated with atrial fibrillation [41]. In a recent study, Baumgarten et al. found that the branch of the kynurenine pathway, leading to the formation of KA, was downregulated in atherosclerotic plaques, and this downregulation was associated with an increased risk of cerebrovascular events [19].

### Mortality

We found that inflammation, in general, was significantly associated with mortality in the unadjusted analyses. In the adjusted analyses, only CRP and neopterin showed added value as predictors compared to traditional parameters such as prior vascular disease and mRS [30]. The lack of association to the remaining biomarkers could again be explained by statistical type II error due to the low sample size and short time of follow-up.

Several inflammatory biomarkers have been found to be independent predictors of all-cause and cardiovascular mortality. Both IL-6 and CRP have been found to be associated with increased mortality [34, 42], and neopterin has been identified as an independent predictor of all-cause and cardiovascular mortality [16]. The PAr-index has been associated with all-cause mortality in patients with coronary artery disease [43].

KA was negatively associated with mortality first after adjusting for inflammation. We did this analysis based on the increasing evidence of KA as a contributor to the body’s anti-inflammatory capacity, as part of a negative feedback loop [19, 20, 44]. KA increases in response to inflammation, and our hypothesis, that a higher level of KA in response to inflammation, measured by neopterin [45] would be associated with a beneficial outcome, was supported as we found a higher value of KA to be associated with reduced risk of mortality when adjusted for inflammation. In fact, both risk estimates were strengthened after adjustment, indicating that they mutually camouflaged some of their individual contributions.

Our current finding is in concordance with KA’s role in vascular disease, amongst others, by inhibiting leukocyte recruitment to atherosclerotic plaques [19]. The result is in contrast to findings of KA being associated with mortality after coronary artery disease when being analysed without adjusting for inflammation [39]. Although exploratory, our results indicate that downregulation of KA might be one of the molecular pathways mediating the hazards of sedentary behaviour in disease progression. This should be further investigated.

### Stroke subtype

We found that patients with cardioembolic strokes at baseline had higher levels of CRP, IL-6, and neopterin at 3 months, indicating a difference in the inflammatory profile between these subgroups. The link between stroke and inflammation has primarily been investigated in all the subtypes combined [14, 46] or in stroke caused by the progression of atherosclerosis [47] or CSVD [48]. The role of inflammation in cardioembolic strokes, in particular, is less studied. However, increasing evidence exists of the importance of inflammation for the presence of atrial fibrillation and the associated cardioembolic risk [7, 8, 49]. It can be argued that concurrent vascular disease might mediate the increased risk of stroke associated with inflammation found in patients with atrial fibrillation. However, Packer argued in a review for the contribution of inflammation as a risk factor independent of vascular disease, as the risk exceeded that predicted by cardiovascular risk factors [50]. Inflammatory biomarkers might be important for identifying those in need of prolonged ECG monitoring to identify paroxysmal atrial fibrillation, and when evaluating stroke risk in patients with atrial fibrillation [7, 38, 50–52]. Altogether, these observations indicate that the stroke subtype is relevant when studying inflammation in stroke and underlines the importance that such studies are powered for stratified analyses.

### Strength and weaknesses

The information about stroke recurrence was based on the Norwegian Stroke Registry, which identifies patients with stroke admitted to hospital. The registry reports coverage of 84–87% in this period [26] and has been shown to be reliable [53]. The registry includes only patients admitted to hospital and where the recurrent stroke has been clinically recognised. Not all patients are admitted to hospital, and not all strokes are clinically overt. Zeestraten et al. found a recurrence rate of 3% based on clinical judgement, while 27% had new lacunas on imaging at the five-year follow-up [54]. These strokes might be more clinically subtle but are still of clinical importance. The use of imaging, such as MRI, could increase the sensitivity of the stroke diagnosis, and this has been included in the updated stroke definition [55]. Hence, we might have underestimated the incidence of ischemic stroke recurrence.

The mean follow-up time of the patients was 2.5 years, and for the patients included at the end of the study, the follow-up was limited to 16 months after the three-month follow-up. Khanevski et al. studied patients with ischemic stroke and transient ischemic attack and found that 5.4% had stroke recurrence after 1 year, 10.7% after 5 years, and 14.2% at the end of the study (mean 5.6 years) [30]. In addition, we had to exclude half of the stroke recurrences because they occurred before the three-month follow-up. The short follow-up and the low number of stroke recurrences in our study increase the risk of a type II error, and in particular negative findings should be evaluated with caution.

To avoid the acute inflammatory response associated with the acute stroke event, we chose the three-month follow-up after the acute stroke as the baseline for this study, assuming the inflammatory biomarkers were not affected by the acute event at that point in time-point [16]. The correlation between the inflammatory biomarkers before and 3 months after is not known. Still, it characterises these subgroups of patients and could be valuable for prospective studies. Stroke subtype is based on clinical judgement of the most likely cause of the stroke in question and may be challenging to determine [6]. Also, several causes of stroke in a single patient are common, i.e. a combination of large artery atherosclerosis, CSVD, and atrial fibrillation. These patients are often included in the category “unknown”.

## Conclusion

Neopterin seems to be a useful prognostic biomarker in stroke populations. The novel finding of a potential protective effect of KA in response to inflammation in disease progression should be investigated further. The results also underline the importance of stroke subtypes when investigating the impact of inflammation in ischemic stroke. The results highlights the importance of inflammation in cerebrovascular disease. Measures should be made to reduce inflammation as a secondary preventive strategy, i.e. by reducing sedentary behaviour. In addition, patients with undetected atrial fibrillation are receiving suboptimal drug treatment, and investigating the usefulness of inflammatory biomarkers to identify these patients could have significant therapeutic consequences for these individuals.

## Supplementary Information

**Additional file 1:.**

## Data Availability

The datasets generated and/or analysed during the current study are not publicly available due to Norwegian regulations and conditions for informed consent but are available from the corresponding author on reasonable request.

## Abbreviations

ATC: Anatomical therapeutic chemical classification
ADL: Activities of daily living
BI: Barthel Index
CI: Confidence interval
CRP: C-reactive protein
CSVD: Cerebral small vessel disease
eGFR: Estimated glomerular filtration rate
ECG: Electro cardiogram
HR: Hazard ratio
IL-6: Interleukin-6
IL-10: Interleukin-10
IQR: Interquartile range
KA: kynurenic acid
LDL: Low density lipoprotein
mRS: Modified Rankin Scale
MRI: Magnetic resonance imaging
NIHSS: National Institute of Stroke Scale
Nor-COAST: Norwegian Cognitive Impairment After Stroke Study
PAr-index: Pyridoxal acid ratio index
PLP: Pyridoxal-5-phosphate
PL: Pyridoxal
PA: Pyridoxal acid
REK: regional ethics committee
SD: Standard deviation
TOAST: Trial of Org 10,172 in Acute Stroke Treatment
WHO: World Health Organization
