# Supplementary material for: Neopterin and kynurenic acid as predictors of stroke recurrence and mortality: a multicentre prospective cohort study on biomarkers of inflammation measured three months after ischemic stroke
Source: BMC Neurol. 2021 Dec 8;21:476. doi: 10.1186/s12883-021-02498-w (PMC8653541; doi:10.1186/s12883-021-02498-w)
Supplement: Supplementary file 1 — Additional file 1:. [file 12883_2021_2498_MOESM1_ESM.docx]

| **Supplementary table 1: Adjusted competing event (death) regression of the associations between biomarkers and the outcomes ischemic stroke recurrence.** | | | | | | | | |
| --- | --- | --- | --- | --- | --- | --- | --- | --- |
|  |  | **Model 1** | | |  | **Model 2** | | |
|  |  | SHR | CI | p |  | SHR | CI | p |
| CRP | | 0.83 | (0.40, 1.71) | 0.613 |  | 0.89 | (0.43, 1.84) | 0.745 |
| IL-6 | | 1.17 | (0.67, 2.04) | 0.578 |  | 1.09 | (0.60, 2.00) | 0.771 |
| Neopterin | | 1.42 | (0.82, 2.47) | 0.215 |  | 1.36 | (0.81, 2.29) | 0.245 |
| PAr-index | | 1.11 | (0.59, 2.10) | 0.748 |  | 1.20 | (0.64, 2.23) | 0.587 |
| Kynurenic acid | | 0.81 | (0.42, 1.58) | 0.544 |  | 0.88 | (0.41, 1.89) | 0.737 |
| Model 1: age, sex, prior cerebrovascular disease, modified Rankin scale at three months, creatinine.  Model 2: As model 1 + TOAST-classification at baseline (TOAST=Trial of Org 10172 in Acute Stroke Treatment)  For CRP n was 212, 8 and 13 for all, recurrent IS and death, respectively.  SHR=subdistribution hazard ratio, CI=confidence interval, CRP= C-reactive protein, IL-6=Interleukin 6, IL-10=Interleukin 10, PAr-index=4-pyridoxic acid:(pyridoxal+pyridoxal-5`-phosphate), KA=kynurenic acid. | | | | | | | | |
